# Multiple Chronic Conditions and Multimorbidity Among Older Adults in Southern Albania: Distribution and Impact on Care Needs, Medication Adherence, and Quality of Life

**DOI:** 10.3390/healthcare14142058

**Published:** 2026-07-09

**Authors:** Brunilda Subashi, Fatjona Kamberi, Erlini Kokalla

**Affiliations:** 1 Department of Biomedicine and Prevention, University of Rome Tor Vergata, Via Montpellier, 1, 00133 Roma, Italy; 2 Scientific Research Center for Public Health, University of Vlora ‘Ismail Qemali’, Lgj. Pavaresia, 9400 Vlore, Albania; 3 Department of Statistical Sciences, Sapienza University of Rome, Piazzale Aldo Moro, 5, 00185 Rome, Italy; 4 Department of Healthcare, Faculty of Health, University of Vlora ‘Ismail Qemali’, Lgj. Pavaresia, 9400 Vlore, Albania

**Keywords:** older adults, multiple chronic conditions, multimorbidity, self-care, medication adherence, quality of life, Albania

## Abstract

**Highlights:**

**What are the main findings?**

- Distribution of chronic conditions: Most older adults had multiple chronic conditions (MCCs), while a smaller subgroup had multimorbidity (MM) characterized by higher clinical and functional complexity.
- Factors associated with MM: MM was strongly linked to advanced age, lower education, polypharmacy, reliance on family care, medication non-adherence, and reduced quality of life, indicating that disease burden alone does not fully capture clinical complexity.

**What are the implications of the main findings?**

- Clinical practice for high-risk groups: Identification of high-risk older adults with MM allows healthcare providers to target interventions such as medication management, adherence support, and functional assistance to improve outcomes.
- Health policy and care planning: Integrated, person-centered care strategies are needed to address both the medical and social dimensions of complex MM, particularly in aging populations with varied educational and functional profiles.

**Abstract:**

**Introduction**: Multimorbidity, defined as the coexistence of two or more chronic conditions, is a major global public health challenge. It exists on a continuum from non-complex to highly complex forms, with increasing complexity associated with greater instrumental activities of daily living (IADL) disability, frailty, and mortality. **Objectives**: This study aims (1) to assess chronic condition complexity (CCC) by distinguishing multiple chronic conditions (MCCs) from multimorbidity (MM); (2) evaluate their distribution and impact on care needs, medication adherence, and quality of life; and (3) examine associations between MM and sociodemographic factors among older adults in Albania. **Methods**: A descriptive, cross-sectional, multicenter observational study was conducted among older adults aged 65 years and older with ≥2 chronic conditions. Data were collected from participants attending six primary health care centers in southern Albania between March and December 2024. Medication adherence and quality of life were assessed using the Simplified Medication Adherence Questionnaire (SMAQ) and the Older People’s Quality of Life Questionnaire (OPQOL-35), both validated for the Albanian population. **Results**: In a sample of 727 older adults with ≥2 chronic conditions, MM was significantly associated with advanced age, polypharmacy, medication non-adherence, and lower educational level (*p* < 0.05). Additional factors, such as reliance on family care and reduced quality of life, were also linked to higher odds of MM, while gender and secondary education showed non-significant associations. **Conclusions**: Although multimorbidity represents a smaller subgroup within MCCs, it is associated with greater clinical and functional complexity, particularly among older adults of advanced age and lower educational level, indicating that disease burden alone does not fully capture clinical complexity.

## 1. Introduction

As global populations age, multimorbidity (MM), the presence of two or more long-term conditions in one individual, is becoming increasingly common and represents one of the greatest global public health challenges facing contemporary health systems [1,2,3]. The number of people living with multiple chronic conditions (MCCs) continues to rise worldwide, placing increasing strain on clinical management and the organization of healthcare services [2,4]. MM exists along a continuum from non-complex (≥2 conditions affecting ≤2 body systems) to complex, which may be classified as moderately complex (3–4 conditions) or highly complex (≥5 conditions); greater complexity and multi-system involvement are associated with worse instrumental activities of daily living (IADL) disability, increased frailty, and higher mortality [5]. Consistent with this framework, Klinedinst et al. categorize MCCs as no MCC (0–1 condition), low MCC (2–4 conditions), and MM (multi-system morbidity with ≥5 conditions) to reflect increasing disease burden and functional outcomes [6]. The broad definition of multimorbidity (≥2 chronic conditions) encompasses a highly diverse group of patients, ranging from those with minimal health impact to those with severe and complex health burdens. These conditions may include physical non-communicable diseases, chronic infectious diseases (such as HIV), and mental health disorders. More recently, the concept of complex multimorbidity has emerged, highlighting that some combinations of conditions are more difficult to manage for healthcare systems, clinicians, and patients than others [3].

MM is frequently accompanied by polypharmacy, as individuals with MCCs commonly require multiple medications, further increasing treatment complexity [7]. Polypharmacy is defined as the regular use of five or more medications, while hyper-polypharmacy refers to the concurrent use of more than ten medications [8]. These patterns are associated with adverse physical and cognitive outcomes, drug–drug interactions, medication nonadherence, and adverse health outcomes (e.g., increased risks of falls, cognitive impairment, hospitalization, and mortality) [9].

Globally, MM is particularly prevalent among older adults, affecting an estimated 55–98% of individuals aged 65 years and older [1,10,11]. As age increases, individuals are more likely to accumulate MCCs [12], and rising life expectancy together with global population aging has further amplified the burden of MM on individuals, healthcare systems, and societies [13]. Consequently, MM is associated with poorer quality of life, greater disability and functional impairment, increased health care utilization, fragmented care, complex treatment regimens, and higher mortality [14].

The burden of MM is especially concerning in low- and middle-income countries (LMICs), where health systems are often less equipped to manage complex chronic care. Although data remain limited, evidence suggests that approximately half of middle-aged and older adults in LMICs experience at least two chronic conditions, with a substantial proportion living with three or more conditions [13]. MM in these settings poses major challenges for governments and health systems and has been consistently associated with poorer quality of life [15].

In Albania, MM is increasingly recognized as a growing health concern, particularly among older adults (<65 years) and women, with several demographic and socioeconomic factors contributing to its prevalence [16]. However, despite this growing burden, empirical evidence on MCCs and MM among older adults (≥65 years) in Albania remains scarce, and existing studies have largely focused on broader population groups or specific clinical contexts.

Given rapid population aging and the rising burden of chronic diseases in Albania, there is a clear need for well-designed studies that assess the distribution of MCCs and MM among older adults and examine their associations with sociodemographic characteristics, medication use, and health-related outcomes (medication adherence, care needs, and quality of life). Addressing this gap is essential to inform health policy, improve care planning, and support the development of integrated, patient-centered approaches for managing chronic condition complexity (CCC) in the Albanian context. Therefore, this study aims (1) to assess chronic condition complexity (CCC) by distinguishing MCCs from MM; (2) evaluate their distribution and impact on care needs, medication adherence, and quality of life; and (3) examine associations between MM and sociodemographic factors among older adults in Albania.

## 2. Materials and Methods

### 2.1. Study Design

A multicenter, cross-sectional study was conducted among older adults aged 65 years and older with ≥2 chronic conditions. Data were collected from participants attending six primary health care centers in the cities of Vlore and Orikum in southern Albania between March and December 2024. Medication adherence and quality of life were assessed using the Simplified Medication Adherence Questionnaire (SMAQ) and the Older People’s Quality of Life Questionnaire (OPQOL-35), both validated for the Albanian population.

### 2.2. Sample and Population

A facility-based consecutive sampling strategy was used to recruit older adults with ≥2 chronic conditions from primary healthcare centers. A total of 727 participants aged 65 years and above met the inclusion criteria and agreed to participate in the study. This approach was considered appropriate given limited availability of population registries in Albania and facilitated timely identification of eligible participants.

Although the use of a facility-based sample may limit generalizability, the inclusion of multiple primary healthcare centers and the relatively large sample size strengthen the robustness and precision of the findings.

The inclusion criteria were: (a) age 65 years or older, (b) a diagnosis of two or more chronic conditions, and (c) residence in urban areas. Participants were excluded if they (a) were younger than 65 years, (b) had only one chronic condition, (c) had significant cognitive, visual, or auditory impairments that could interfere with reliable participation in the study.

**Figure 1:**
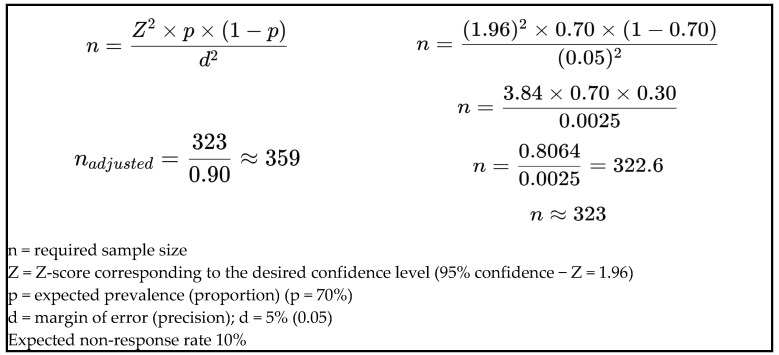
Sample size calculation.

Sample size calculation: The required sample size was calculated using the formula for estimating a population proportion, assuming a 95% confidence level, a 5% margin of error, and an expected prevalence of 70% for MCCs among older adults. Based on this calculation, the minimum required sample size was 323 participants, which was increased to 359 to account for an anticipated 10% non-response rate (Figure 1).

Statistical power and precision: Although the minimum required sample size was 323–359 participants, the final sample of 727 older adults was included to increase the precision of prevalence estimates, improve the reliability of subgroup analyses, and enhance the statistical power to detect associations with sociodemographic and clinical variables.

Categorization of the sample and definition of subgroups: All participants included in the study were classified into two groups (MCCs vs. MM) based on the number of chronic conditions they had. Participants with 2–4 conditions were classified into the MCC group, while those with ≥5 conditions were classified into the MM group, following the classification approach described by Klinedinst [6]. The number of chronic conditions was obtained through participant self-report. To minimize reporting bias, structured questioning was used, including prompts about physician-diagnosed conditions and the number of chronic conditions, and responses were cross-checked for consistency with reported medication use.

To identify a higher-complexity (high-risk) subgroup, participants were further stratified based on clinical and functional criteria. Clinical complexity was assessed using indicators such as the number of chronic conditions, number of medications, and medication adherence, while functional complexity was evaluated through care needs and quality of life.

A composite clinical and functional complexity score was developed using five indicators: number of chronic conditions, polypharmacy, medication non-adherence, reliance on family care and reduced quality of life. Each variable was scored as 0 (absence) or 1 (presence), resulting in a total score ranging from 0 to 5. Participants scoring 0–2 were classified as having lower complexity (MCCs), whereas those scoring ≥ 3 were classified as having higher complexity (MM).

This score was developed for the purposes of this study, drawing on established indicators of clinical and functional complexity. The inclusion of the number of conditions reflects overall disease burden, while the additional variables capture treatment burden and functional impact. Detailed information is presented in Table 1.

Instruments used: The instruments used in this study were the Simplified Medication Adherence Questionnaire (SMAQ), which comprises 7 items, and the Older People’s Quality of Life Questionnaire (OPQOL-35), which comprises 35 items. Both instruments have been validated and demonstrated to be reliable in the Albanian population.

The main outcome variable was multimorbidity status (MCCs vs. MM).

The independent variables included sociodemographic factors: age, gender, education; clinical factors: number of medications and medication adherence; and functional factors: care needs and quality of life.

### 2.3. Institutional Approval and Study Authorization

The study was conducted in accordance with the principles of the Declaration of Helsinki. Ethical and administrative approval for study implementation and use of the research instruments in six Primary Health Care Centers in the cities of Vlora and Orikum was granted by the Regional Directorate of the Vlora Healthcare Services Operator (protocol code no. 358/1, approval date 28 February 2024). Information is presented in Supplementary Section S1.

### 2.4. Participant Information and Informed Consent

Informed consent was obtained from all participants prior to study participation. All information collected was treated with strict confidentiality and used exclusively for scientific purposes, in accordance with the principles of the Declaration of Helsinki.

Before providing informed consent, participants received two documents: (1) a Participant Information Sheet and (2) a Participant Informed Consent Form. The Participant Information Sheet described the study’s purpose, participant selection criteria, study procedures, the importance of participation, and potential benefits (Supplementary Section S2). The Participant Informed Consent Form documented participants’ voluntary agreement to participate after confirming their understanding of the study objectives, procedures, and their rights, including the right to withdraw at any time without consequences or the need to provide justification (Supplementary Section S3). Participant anonymity and data confidentiality were fully assured, and all information was used solely for research purposes.

### 2.5. Data Collection

Data were collected using the Simplified Medication Adherence Questionnaire (SMAQ) and the Older People’s Quality of Life Questionnaire (OPQOL-35), administered via Google Forms. Data collection was conducted by previously trained personnel.

### 2.6. Data Analysis

Data were analyzed using SPSS version 22 and Jamovi version 2.3.28. Descriptive and bivariate analyses were performed, with quantitative variables summarized using measures of central tendency and dispersion and categorical variables presented as absolute and relative frequencies. Bivariate associations were assessed using the chi-square test, where appropriate. A *p*-value < 0.05 was considered statistically significant.

## 3. Results

### 3.1. Characteristics of the Sample

The characteristics of the study sample included age category, gender, educational level, living arrangement (living alone), care needs, number of chronic conditions, number of medications, number of hospitalizations in the previous year, and primary healthcare center. These variables were selected because they capture the key sociodemographic, clinical, functional, and healthcare utilization domains relevant to MM and health outcomes in older adults. Age, gender, and educational level represent fundamental sociodemographic determinants of health. Living alone and care needs reflect functional status and the availability of social support, which are particularly important in older populations. The number of chronic conditions and number of medications capture clinical complexity and treatment burden, including MM and polypharmacy. Hospitalizations in the previous year serve as an indicator of healthcare utilization and overall disease severity. Finally, the primary healthcare center was included to account for potential variability across study sites. Detailed information is presented in Table 2.

Table 2 presents the sociodemographic, clinical, and functional characteristics of the study sample. Most participants were youngest-old adults (65.9%) and female (56.3%). Nearly half had had secondary education (45.5%), and the majority did not live alone (83.3%). Slightly more than half of participants received care from family (53.9%), while 46.1% reported self-care.

Regarding clinical characteristics, most participants (96.3%) were classified as having MCCs (2–4 chronic conditions), while a small proportion (3.7%) met the criteria for MM (≥5 chronic conditions). The majority had two chronic conditions (65.1%), followed by three chronic conditions (23.2%). Most participants (80.3%) used <5 medications, with 4 medications being the most common regimen (29.3%). The mean number of medications was 3.43 ± 1.31, and 19.7% of participants met criteria for polypharmacy (≥5 medications). More than half of participants (56.1%) reported no hospitalizations in the previous year. Participants were recruited from multiple healthcare centers, with the largest proportion attending Healthcare Center No. 1 (30,0%) and similar proportions from the other centers.

### 3.2. Identifying the Higher-Complexity Subgroup

To identify a higher-complexity (high-risk) subgroup, participants were further stratified based on clinical and functional criteria. Clinical complexity was assessed using indicators such as the number of chronic conditions, polypharmacy (≥5 medications) and medication non-adherence, while functional complexity was evaluated through reliance on family care and quality of life.

As shown in Table 3, most participants were classified into the lower-complexity group (MCCs), accounting for 96.7% (*n* = 703) of the sample. In contrast, only 3.3% (*n* = 24) were categorized as having higher clinical complexity (MM), representing a relatively small high-risk group.

The two classification approaches (Table 2 and Table 3) yielded highly consistent results, with MCCs comprising 96.3–96.7% of the sample and MM representing 3.3–3.7%. Minor differences in subgroup size likely reflect variations in classification criteria and do not affect the overall distribution pattern, which remains consistent across both classifications.

### 3.3. Logistic Regression of Factors Associated with Chronic Condition Complexity (MCCs vs. MM)

Participants with MCCs were categorized based on the total number of conditions following Klinedinst et al. (2022), who classify MCCs as (a) no MCC (0–1 condition), (b) low MCC (2–4 conditions), and (c) multi-system morbidity (MM ≥ 5 conditions). This approach aligns with common methods in multimorbidity research to capture differences in complexity and health outcomes [6].

We examined the association of MM with age, gender, educational level, number of medications, care needs, medication adherence, and quality of life. Due to the limited number of MM cases (*n* = 27), the multivariable analysis was restricted to a small number of theoretically relevant factors (2–3 key factors) to maintain an acceptable events-per-variable ratio and minimize overfitting. Additional variables were examined in univariable analyses. Detailed information is presented in Table 4.

The analysis identified several factors associated with the likelihood of being classified into the MCC group compared to the MM group. In the reduced multivariable model (3 factors; EPV = 9, acceptable model stability), age, number of medications, and medication adherence emerged as significant independent factors. An EPV close to 10 is generally considered indicative of acceptable model stability, suggesting that the estimated associations are reasonably reliable, although still potentially sensitive to sample-specific variation [17,18]. Individuals in the oldest-old category had significantly higher odds of MM compared with the youngest-old (OR = 4.73, 95% CI 1.71–13.07, *p* = 0.003). Similarly, participants taking ≥ 5 medications were over six times more likely to have MM than those taking fewer medications (OR = 6.15, 95% CI 2.97–14.60, *p* < 0.001), representing the strongest association observed. In addition, non-adherence to medication was linked to nearly fivefold higher odds of MM compared to adherence (OR = 4.86, 95% CI 1.22–19.28, *p* = 0.025).

In contrast, the univariable analyses demonstrated high stability, with EPV values of 27, substantially exceeding commonly recommended thresholds. These models identified several additional variables associated with the outcome. Highest educational level appeared to have a protective effect, with significantly lower odds compared to lower education (OR = 0.19, 95% CI: 0.04–0.86, *p* = 0.031), while secondary education showed a borderline association (OR = 0.47, *p* = 0.068). Participants receiving care from family members had higher odds of MM than those managing self-care (OR = 2.52, 95% CI: 1.05–6.05, *p* = 0.038), and those with lower quality of life were also more likely to have MM (OR = 2.99, 95% CI: 1.16–7.69, *p* = 0.023). Gender was not significantly associated with MM (*p* = 0.138).

From a modeling perspective, the use of a reduced multivariable model helps mitigate overfitting by limiting the number of predictors relative to the number of events. However, the EPV of 9 remains slightly below more conservative thresholds (e.g., EPV ≥ 10–15), indicating that some risk of overfitting persists. This may lead to optimistic effect estimates and reduced reproducibility in other samples [18,19]. Nevertheless, the consistency of key predictors such as age, polypharmacy, and medication adherence with the existing literature supports their plausibility across settings.

Overall, the findings suggest that advanced age, polypharmacy, and medication non-adherence are the most important independent factors of MM. At the same time, educational level, care needs, and quality of life may contribute to the risk profile but require further investigation in larger, well-validated models to confirm their independent effects and improve the generalizability of these results.

## 4. Discussion

This study examined CCC among older adults in southern Albania by distinguishing MCCs from MM, assessing their distribution and impact on care needs, medication adherence, and quality of life, and evaluating associations between MM and sociodemographic factors.

Chronic condition complexity and subgroup distribution

In relation to the first objective, the findings indicate that having MCCs was highly prevalent, affecting 96.7% of participants, whereas only 3.3% were classified as having MM. This contrast reflects the distinction between a broad disease-count definition and a more restrictive, multidimensional conceptualization of clinical complexity.

The relatively low proportion of MM differs from traditional prevalence estimates but is consistent with emerging evidence on complex MM, which recognizes that not all individuals with MCCs experience the same level of clinical burden [20]. By incorporating treatment burden and functional impairment into the classification, this study identifies a smaller, more clinically meaningful high-risk subgroup. This approach aligns with the recent literature suggesting that simple disease counts are insufficient to capture the heterogeneity and true care needs of multimorbid populations [20,21].

Impact on care needs, medication adherence, and quality of life

Regarding the second objective, the findings demonstrate that higher clinical complexity is associated with greater reliance on family care, poorer medication adherence, and lower quality of life. These results are consistent with previous studies showing that complex MM is linked to increased dependence and reduced quality of life [22,23,24,25].

Medication non-adherence was significantly associated with MM, supporting evidence that treatment complexity is a major barrier to adherence. As highlighted in a systematic review by Yap et al. (2016), polypharmacy and complex therapeutic regimens can significantly reduce adherence among older adults with MM [26]. This relationship is likely bidirectional, as poor adherence may further exacerbate disease progression and increase clinical instability.

Similarly, the association between MM and lower quality of life is well established. Fortin et al. (2004) demonstrated that quality of life declines with increasing disease burden, particularly when functional limitations are present [22]. The observed reliance on family care among participants with MM further reflects the functional and social consequences of higher clinical complexity, emphasizing the need for integrated care models that address both medical and social dimensions.

Associations with sociodemographic and clinical factors

In line with the third objective, several key factors of higher clinical complexity were identified. Advanced age was strongly associated with MM, with oldest-old participants having significantly higher odds compared to the youngest-old. This finding is consistent with extensive evidence indicating that aging is a major driver of both MM and its progression to more complex states [23,27,28,29,30].

Polypharmacy emerged as one of the strongest factors, with participants taking ≥5 medications having over six times higher odds of MM. This is consistent with a large body of literature identifying polypharmacy as both a marker and driver of MM [26,31,32,33]. While often clinically necessary, polypharmacy increases treatment burden and the risk of adverse outcomes, including hospitalization and mortality, thereby reinforcing its central role in defining clinical complexity [31].

Medication non-adherence was also significantly associated with MM, with non-adherent individuals showing substantially higher odds of higher complexity. This finding aligns with previous research indicating that complex medication regimens negatively influence adherence and, consequently, health outcomes [33].

Educational level demonstrated a protective effect, with highest education significantly associated with lower odds of MM. This supports existing evidence on socioeconomic gradients in health, where individuals with highest education tend to have better health literacy, improved self-management, and greater access to healthcare resources [34,35]. In contrast, reliance on family care was associated with increased odds of MM, reflecting greater functional dependency and care needs. Similar patterns have been reported in other populations, where multimorbid individuals are more likely to require informal caregiving due to declining independence [36].

Gender was not significantly associated with MM in this study, although a non-significant trend toward higher odds among women was observed. This is consistent with mixed findings in the literature, where gender differences often attenuate after adjusting for age and socioeconomic factors [2].

Context within Albania

Evidence on MM in Albania remains limited. Existing studies have primarily focused on general non-communicable disease risk factors or conceptual discussions, with limited analysis of clinical complexity and its determinants among older adults. For example, studies by Collaku et al. emphasize the clinical complexity of MM and the need for integrated care but lack empirical data on its distribution and associated factors [37], while national surveys, such as those by Kraja et al., focus on risk factors in adults (≥35 years old) rather than patterns of MM [38]. The present study contributes to filling this gap by providing empirical evidence on the distribution and determinants of complex MM among older adults in Albania.

## 5. Limitations

Several limitations should be acknowledged. The facility-based design may limit generalizability, and the use of self-reported data for chronic conditions may introduce recall bias, although structured questioning and consistency checks were applied. Additionally, the small MM subgroup may have limited statistical power for detecting weaker associations.

## 6. Conclusions

In this study, MCCs were highly common among older adults, while a smaller proportion were classified as having MM. This subgroup was characterized by the presence of polypharmacy, medication non-adherence, reliance on family care, and reduced quality of life and was strongly associated with advanced age and lower educational level. These findings highlight the distinction between disease burden and clinical complexity, demonstrating that not all individuals with MCCs experience the same level of health-related challenges.

## 7. Implications for Research, Practice, and Policy

Further population-based and longitudinal studies are needed to validate the distinction between MCCs and higher-complexity MM and to examine their progression over time. Future research should also refine multidimensional measures of clinical complexity and evaluate interventions targeting polypharmacy, medication adherence, and quality of life.

The findings support the need for person-centered, integrated care approaches that go beyond disease count. Routine assessment of polypharmacy, medication adherence, care needs, and quality of life should be incorporated into primary care, alongside medication review and adherence support strategies.

Policies should strengthen primary care and promote integrated care models for multimorbidity. Developing national guidelines, supporting multidisciplinary care, and enhancing support for informal caregivers are essential to address the growing burden of clinical complexity among older adults.

## Figures and Tables

**Table 1:** Clinical and functional criteria used to define the composite complexity score.

Clinical Criteria	Functional Criteria
Number of conditions	Reliance on family care
0 = 2–4 conditions	0 = independent
1 = ≥5 conditions	1 = dependent
Polypharmacy	Quality of life
0 = <5 medications	0 = higher QoL
1 = ≥5 medications	1 = lower QoL
Medication non-adherence	**Participants scoring classifications**
0 = adherent	Lower complexity (MCCs) = 0–2 score
1 = non-adherent	Higher complexity (MM) ≥3 score

Note: QoL—Quality of Life; MCCs—Multiple Chronic Conditions; MM—Multimorbidity. Participants scoring classifications: This section of the table shows the classification of participants based on total points according to the above categories (1. number of conditions, 2. polypharmacy, 3. medication non-adherence, 4. reliance on family care, and 5. quality of life).

**Table 2:** Demographic variables.

Descriptives		Counts	% of Total
Age category	Youngest-old	479	65.9
	Middle-old	199	27.4
	Oldest-old	49	6.7
Gender	Female	409	56.3
	Male	318	43.7
Education level	Basic education	240	33.0
	Secondary education	331	45.5
	Highest education	156	21.5
Living alone	Yes	121	16.7
	No	605	83.3
Care needs	Self-care	335	46.1
	Care from family	392	53.9
Number of chronic conditions	MCCs	700	96.3
	MM	27	3.7
Number of medications	<5 medications	584	80.3
	≥5 medications	143	19.7
Number of hospitalizations in previous year	0	408	56.1
	1–2	265	36.5
	3–4	54	7.4
	4+	0	0.0
Healthcare center	HCC No. 1	218	30.0
	HCC No. 2	102	14
	HCC No. 3	105	14.4
	HCC No. 4	101	13.9
	HCC No. 5	101	13.9
	HCC No. 6	100	13.8

Note: MCCs—Multiple Chronic Conditions; MM—Multimorbidity; HCC—Healthcare Center.

**Table 3:** Identifying the higher-complexity (high-risk) subgroup.

Subgroups	Counts	% of Total
MCCs	703	96.7
MM	24	3.3

**Table 4:** Logistic Regression Analysis of Factors Associated with MM (*n* = 27).

Factor(s)	OR (95% CI)	*p*-Value	Model Type	No. of Factors	EPV	Stability
Age						
Oldest-old—Youngest-old	4.73 (1.71–13.07)	0.003	Multivariable (reduced)	3	9	Acceptable
Number of medications						
≥5 medications—<5 medications	6.15 (2.97–14.60)	<0.001	Multivariable (reduced)	3	9	Acceptable
Medication adherence						
Non-adherent—Adherent	4.86 (1.22–19.28)	0.025	Multivariable (reduced)	3	9	Acceptable
Gender						
Female—Male	1.89 (0.82–4.34)	0.138	Univariable	1	27	Very stable
Education level						
Secondary education—Lower education	0.47 (0.21–1.06)	0.068	Univariable	1	27	Very stable
Highest education—Lower education	0.19 (0.04–0.86)	0.031	Univariable	1	27	Very stable
Care needs						
Care from family—Self-care	2.52 (1.05–6.05)	0.038	Univariable	1	27	Very stable
Quality of life						
Low QoL—High QoL	2.99 (1.16–7.69)	0.023	Univariable	1	27	Very stable

Note: Estimates represent the log odds of “MCC vs. MM = MM” vs. “MCC vs. MM = MCC”.

## Data Availability

The datasets presented in this article are not readily available because the data are part of an ongoing study. Requests to access the datasets should be directed to corresponding author.

## Supplementary Materials

The following supporting information can be downloaded at: https://www.mdpi.com/article/10.3390/healthcare14142058/s1. Supplementary Section S1: Institutional Approval and Study Authorization. Supplementary Section S2: Participant information sheet. Supplementary Section S3: Participant Consent Form.

## Abbreviations

The following abbreviations are used in this manuscript:

NCDs	Non-Communicable Diseases
CCCs	Complex Chronic Conditions/Chronic Conditions Complexity
MCCs	Multiple Chronic Conditions
MM	Multimorbidity
EPV	Event-Per-Variable
