# Supplementary material for: Multiple Chronic Conditions and Multimorbidity Among Older Adults in Southern Albania: Distribution and Impact on Care Needs, Medication Adherence, and Quality of Life
Source: Healthcare (Basel). 2026 Jul 9;14(14):2058. doi: 10.3390/healthcare14142058 (PMC13409679; doi:10.3390/healthcare14142058)
Supplement: Supplementary file 1 [file healthcare-14-02058-s001.zip › Description of content Section S1.pdf]

1. S1. INSTITUTIONAL APPROVAL AND STUDY AUTHORIZATION

OFFICIAL APPROVAL FOR DATA COLLECTION IN PRIMARY HEALTH CARE CENTERS IN VLORA AND ORIKUM

The document below provides official approval for the conduct of data collection in six primary health care centers in the cities of Vlora and Orikum, related to the study entitled "Self-*care, medication adherence, and quality of life in older adults with multiple chronic conditions"*. The approval was granted by the Regional Directorate of the Vlora Healthcare Services Operator (P*rotocol Code* no. 358/1, approval date 28 February 2024).

The document confirms authorization to carry out data collection within primary health care centers under the Vlore Local Health Care Unit and specifies that the research activities will not interfere with the routine delivery of health services. The approval applies to the following facilities:

1. Health Care Center No. 1 Vlore
2. Health Care Center No. 2 Vlore
3. Health Care Center No. 3 Vlore
4. Health Care Center No. 4 Vlore
5. Health Care Center No. 5 Vlore
6. Orikum Health Care Center Vlore

REPUBLIKA E SHQIPËRISË
MINISTRIA E SHËNDETËSISË DHE MBROJTJES SOCIALE
OPERATORI I SHËRBIMEVE TE KUJDESIT SHËNDETËSOR
DREJTORIA RAJONALE VLORË
DREJTORIA E SHËRBIMIT PARËSOR

Nr. 358/1 Prot. Vlorë, më 28.02 2024

Lënda: Miratim kërkese

UNIVERSITETIT "ISMAIL QEMALI" VLORË
FAKUTETIT TË SHËNDETIT
Znj. Brunilda Subashi

Për dijeni: **NJËSISË VENDORE TË KUJDESIT SHËNDETËSOR VLORË**

Në zbatim të V.K.M nr.419, datë 4.7.2018 "Për krijimin, mënyrën e organizimit dhe të funksionimit të Operatorit të Shërbimeve të Kujdesit Shëndetësor", të Ligjit nr.7961, datë 12.7.1995 "Kodi i punës i Republikës së Shqipërisë" i ndryshuar, të Urdhërit nr.18 nr.321 prot., datë 17.01.2019 të MSHMS "Miratim i Rregullores së Brendshme të Operatorit të Shërbimeve të Kujdesit Shëndetësor". Ligjin Nr.10107, datë 30.3.2009 "Për Kujdesin Shëndetësor në Republikën e Shqipërisë, Urdhrin Nr. 413 datë 13.07.2022 të MSHMS "Për Miratimin e Statutit të Qendrës Shëndetësore", Ligjin Nr. 9887, datë 10.03.2008 "Për mbrojtjen e të dhënave personale", si dhe bazuar në shkresën tuaj me lëndë " kërkësë për leje" protokolluar pranë DROSHKSH Vlorë me nr. 358 prot, datë 20.02.2024.

Miratoj:

Kërkesën për leje mbi proçesin e grumbullimit të të dhënave në Qendrat Shëndetësore pranë NJVKSH Vlorë në kuadër të studimit me titull "Vetëkujdesi, aderenca ndaj mjekimit dhe cilësia e jetës tek të moshuarit me sëmundje kronike të shumëfishta", duke mos cënuar shërbimin shëndetësor në qendrat shëndetësore si më poshtë:

1. Qendra Shëndetësore Nr.1 Vlorë
2. Qendra Shëndetësore Nr.2 Vlorë
3. Qendra Shëndetësore Nr.3 Vlorë
4. Qendra Shëndetësore Nr.4 Vlorë
5. Qendra Shëndetësore Nr.5 Vlorë
6. Qendra Shëndetësore Orikum Vlorë

Faleminderit!

DREJTOR

Lorene Rexho

Adresa: Lagjia "15 Tetori", Rruga "Mitaq Sallata", Vlorë tel. 033 22 2647, e-mail:DROSHKSH.Vlore@oshksh.gov.al
