# Supplementary material for: Multiple Chronic Conditions and Multimorbidity Among Older Adults in Southern Albania: Distribution and Impact on Care Needs, Medication Adherence, and Quality of Life
Source: Healthcare (Basel). 2026 Jul 9;14(14):2058. doi: 10.3390/healthcare14142058 (PMC13409679; doi:10.3390/healthcare14142058)
Supplement: Supplementary file 1 [file healthcare-14-02058-s001.zip › healthcare-4169396-supplementary-Sections S2 and S3.pdf]

S2. PARTICIPANT INFORMATION SHEET

1. **S2. Participant information sheet**

Informing study subjects about the purpose of the study, why they have been selected to participate in this study, why their participation is important, and what their benefits from this study will be.

INFORMATION SHEET

Information sheet for: Doctoral thesis on the topic: '*Self-care, adherence to medication and quality of life in the elderly with multiple chronic conditions*'.

You have been selected to be part of this research study.
Before you decide whether or not to participate, it is important that you understand why this study is being conducted and who is involved.
Please read the following information carefully.

What is the purpose of the study?
I, the undersigned Brunilda Subashi, lecturer at the University of Vlora "Ismail Qemali", Faculty of Public Health, currently a doctoral student in Nursing Sciences and Public Health at the Faculty of Medicine and Surgery, University 'Tor Vergata' Rome, am conducting a study on self-care, adherence to medication and quality of life in older adults with multiple chronic conditions. The results of the study will benefit all nurses and the elderly over 65 years of age in general. The results will contribute to the field of health policy-making regarding the promotion of a healthy lifestyle as well as the prevention and management of multiple chronic conditions in older adults.

Why were you chosen to participate in this study?
You were randomly selected as all older adults aged 65 years and above can be part of the study regardless of their socio-economic and educational level.

Am I obligated to participate in this study?
It is up to you whether or not you will be part of this study. Participation is voluntary. If you decide to participate, you will be asked to sign a consent form. If you do not wish to participate, you can withdraw at any time without giving a reason.

What will I need to do?
If you decide to participate in the study, you will need to take a few minutes to complete a questionnaire (anonymous) regarding the topic of the study.

Confidentiality (Data Storage)
All information collected will be anonymous and confidential. Your personal data will be stored in accordance with the Law on the Protection of Personal Data.

What will be done with the information collected?
All data that can identify you will be removed during data storage.
They will be stored by the researcher and data analyst for the purpose of the study.
Thank you for taking the time to read this information letter.

With respect,
Brunilda Subashi

S3. PARTICIPANT INFORMED CONSENT FORM

2. S3. Participant Consent Form

Informing study subjects about giving consent to participate in the study voluntarily, assuring them of the anonymity and confidentiality of their data, as well as the right to withdraw from the study at any time.

INFORMED CONSENT FORM

Informed consent form for: Doctoral thesis study on the topic: '*Self-care, medication adherence and quality of life in older adults with multiple chronic conditions*'.

- I have read and understood the information sheet about the study. ☐
- I will give you the opportunity to ask me questions about this study. ☐
- I agree to be part of this study. ☐
- I can withdraw from the study at any time and without explaining the reasons. ☐
- I understand that the information I will provide may be included in publications, on websites and for other reporting purposes and I will not be identifiable. ☐
- I agree that the data will be stored by the study author and the data analyst with the terms that are specified regarding data confidentiality ☐
- I agree to sign any materials related to the study. ☐

Participant's name	Signature	Date
Researcher	Signature	Date
